# Network-based integration of metabolomics data from large-scale repositories

**DOI:** 10.1007/s11306-026-02507-4

**Published:** 2026-07-15

**Authors:** Cecilia Wieder, Eloisa Rocha Liedl, Thomas Payne, Ozgur Yurekten, Callum Martin, Felix Xavier Amaladoss, Noemi Tejera, Wanchang Lin, Yasin El Abiead, Pieter Dorrestein, Claire O’Donovan, Juan Antonio Vizcaíno, Warwick Dunn, Timothy Ebbels

**Affiliations:** 1 Section of Bioinformatics, Division of Systems Medicine, Department of Metabolism, Digestion & Reproduction, Imperial College London, Hammersmith Hospital, Du Cane Road, London, W12 0NN UK; 2 European Molecular Biology Laboratory, European Bioinformatics Institute (EMBL-EBI), Wellcome Trust Genome CampusHinxton, Cambridge, CB10 1SD UK; 3 Department of Biochemistry, Cell and Systems Biology, Centre for Metabolomics Research, Institute of Systems, Molecular and Integrative Biology, University of Liverpool, Liverpool, L69 7ZB UK; 4 Department of Natural Sciences and Sustainable Resources, Institute of Analytical Chemistry, BOKU University, Vienna, Austria; 5 Skaggs School of Pharmacy and Pharmaceutical Sciences, University of California, La Jolla, San Diego, CA 92093 USA

**Keywords:** Public data reuse, Repositories, Data integration, Networks, Harmonised annotation

## Abstract

**Introduction:**

Public metabolomics data repositories such as MetaboLights and Metabolomics Workbench host rapidly growing volumes of raw data, processed results, and metadata. As data deposition becomes a prerequisite for funding and publication, there is an increasing need for tools that enable integration and joint reanalysis of datasets across studies to maximise reuse and reproducibility.

**Objectives:**

This study aims to enable large-scale integrative meta-analysis of public metabolomics data, exploiting harmonised metabolite annotations to identify robust multi-study metabolite and pathway signatures and to provide global visual overviews of repository content.

**Methods:**

We developed a network-based integration framework operating at both the study (dataset) level and the metabolite or pathway level. Metabolite-level meta-networks integrate studies with shared biological context using co-occurrences of differential metabolites represented as bipartite graphs. Study-level networks compare observed metabolites for overall repository exploration. Networks can be explored interactively using a dedicated Python Dash app available at https://github.com/EloisaRL/Metabolomic-data-analysis-app/tree/main.

**Results:**

As an example, the approach was applied to six COVID-19 plasma datasets from MetaboLights generated using LC-MS and NMR. Ten metabolites were identified as differential in at least three studies, including consistently up-regulated pyroglutamic acid, in agreement with the literature. Pathway-level networks provided an overview of shared biological processes across studies. A global network of 1,181 studies in Metabolomics Workbench demonstrated clustering by assay coverage and associated metadata, as expected.

**Conclusion:**

Network-based integration of harmonised metabolomics data enables robust cross-study analyses and highlights the critical importance of standardised annotation pipelines. Such approaches enhance the reuse, reproducibility, and impact of public metabolomics datasets, accelerating biological discovery.

**Supplementary Information:**

The online version contains supplementary material available at https://doi.org/10.1007/s11306-026-02507-4.

## Introduction

As metabolomics approaches become increasingly popular as part of high-throughput omics experiments, there is an urgent need for tools facilitating sharing, access, and re-use of both raw and processed datasets (Stancliffe & Patti, 2022; Wang et al., 2016; Witting, 2023). The public availability of metabolomics data is critical for the research community, enabling results to be reproduced, new methods and tools to be benchmarked, and importantly facilitating integration of multiple studies for reproducibility in science. MetaboLights (Yurekten et al., 2024), Metabolomics Workbench (Sud et al., 2016), and GNPS (Wang et al., 2016) are among the largest repositories available for metabolomics data deposition, containing thousands of publicly available studies and associated metadata (El Abiead et al., 2025). As an example, the MetaboLights repository at the European Bioinformatics Institute alone contains over 15,000 submitted studies (Yurekten et al., 2024), and its content continues to grow rapidly since becoming the recommended metabolomics data repository of many leading scientific journals. Study data in MetaboLights encompasses over 7,000 different organisms (taxonomy IDs) and organism parts, highlighting the diverse origins of metabolomics data collected.

In recent years, both MetaboLights and Metabolomics Workbench have implemented file formats for processed metabolomics data and metadata, in an effort to increase the quality and re-use of public datasets. This, alongside standardised metabolite nomenclature (i.e. the use of ChEBI (Hastings et al., 2016; Malik et al., 2026) or RefMet (Fahy & Subramaniam, 2020) identifiers and names) provides new opportunities for the integrated analysis of multiple datasets. Currently, Metabolomics Workbench offers several tools for exploration of multiple datasets, for example MetStat computes an ANOVA p-value and relative standard deviation for each metabolite in the selected datasets (Sud et al., 2016). The Amanida (Llambrich et al., 2022) package uses the Fisher method to combine p-values and fold-changes from different metabolomics datasets, bypassing the need for obtaining the processed data matrix for each study. However, by only using summary statistics, deeper insights from the raw or processed data may be overlooked. With the increasing volume of data available in repositories, there is an emerging need for tools which can integrate, summarise, and visualise the data, ideally directly compatible with file formats and conventions supported by these resources and associated communities. Network-based models in particular provide an intuitive visual representation of data (for example metabolites, pathways, or studies) and the relationships between them. iDMET (Matsuta et al., 2022) is a network-based meta-analysis approach in which pairs of differential metabolites are connected based on an odds-ratio, however this relies on a specific set of cancer related studies and primarily uses summary statistics rather than using the full scope of the processed metabolite data.

MetaboLights, Metabolomics Workbench, and GNPS have implemented the use of controlled vocabularies (CVs) or ontologies along with submission guidelines in an effort to ensure that the submitted data are of sufficient quality and the resulting data and file formats are interoperable. Despite this, many submitters omit key pieces of study or sample metadata. For example, Harrieder et al. (Harrieder et al., 2022) found that in 70% of cases, chromatographic related metadata (i.e. data relating to the experimental setup e.g. column name, diameter, flow rate, etc.) was incomplete in MetaboLights and Metabolomics Workbench studies. Limited metadata is one of the major bottlenecks that has hindered the re-use and re-analysis of publicly available metabolomics data, but also it is often the main limitation when re-using data coming from public data resources in other omics disciplines (Griss et al., 2015). However, integrative or meta-analysis of metabolomics datasets can be more complex than in other omics, primarily due to the diversity in analytical platforms and metabolites profiled, as well as file formats and metabolite nomenclature used by different vendors, software, and workflows (Koistinen et al., 2023; Liyanage et al., 2024; Llambrich et al., 2022; Patti et al., 2012; Temprosa et al., 2022).

Here, we demonstrate a pipeline for network-based integration of publicly available metabolomics data from MetaboLights and Metabolomics Workbench. In particular, we emphasise the importance of harmonised metabolite annotations in public data repositories, which are the cornerstone of integrative analyses. Inspired by the human disease-gene network presented by Goh *et al. (*Goh et al., 2007) which represents known disease-gene interactions as a bipartite graph, we applied a similar graph-based representation to visualise metabolomics data, with either metabolites or studies as nodes. We constructed both a metabolite-level meta network, where multiple studies sharing a similar outcome/phenotype variable are integrated in terms of their common differential metabolites (i.e. a network-based meta-analysis), and a study-level network, in which hundreds of datasets are integrated in terms of the metabolites profiled. Both approaches are designed to facilitate visualisation and network analysis of publicly available metabolomics data. The metabolite-level meta network is appropriate for a smaller number of similar datasets and enables the identification of cross-study metabolite signatures, for example to answer questions such as ‘which metabolites are consistently differential in COVID-19 plasma samples?’. The study-level network enables users to obtain a visual overview of the entire repository and its assay/metadata coverage. Leveraging publicly available raw metabolomics data in repositories, we can advance upon previous methods, including the ability to harmonise metabolite annotations using the Liverpool Annotation of metabolites using Mass sPectrometry (LAMP) annotation approach (Lin, 2025) as well as producing a pathway-level meta network which integrates the metabolomics data at the pathway level. All the analyses presented here can be replicated by practitioners on their own and public data using a freely available interactive Python Dash app provided as a companion to this paper (see Figure S2).

Overall, this proof of principle study demonstrates the power of network-based approaches to perform multi-study integration across and within metabolomics repositories. Case-studies reveal cross-study metabolite and pathway co-occurrences, as well as an overview of database-wide assay coverage.

## Methods

### Study selection and quality control

For the metabolite and pathway-level meta network examples, human studies in public repositories were prioritised based on the disease (i.e. COVID-19), as well as maximising the number of samples (*n* > 10 per group), the study design (two groups), and the number of annotated metabolites provided by the submitter (20+). Study data (abundance tables and sample metadata) were downloaded manually in October 2024 directly from the MetaboLights or Metabolomics Workbench web interface for each study. The *N* selected studies will be denoted below by the set $\:S=\left\{{s}_{1},\:{s}_{2},\:\dots\:,\:{s}_{N}\right\}$ and are listed in Table 1 (for the metabolite/pathway-level meta network) and Table S1 (for the Metabolomics Workbench bipartite network). The following operations were performed on the sample-by-metabolite matrices$\:\:X$ (size *n*x*M*) : removal of columns with over 50% missing values, K-nearest neighbours imputation (sklearn KNNImputer), $\:{log}_{2}$ transformation, unit-variance scaling (sklearn StandardScaler), outlier removal, and principal component analysis (PCA). Outliers were detected using a range of techniques (PCA scores & residuals, Hotelling’s T^2^ distance, feature Z-scores) and removed where their presence clearly occluded group separation. We emphasise that our integration workflow does not depend on any particular pre-processing or outlier detection procedure, as long as differential metabolites can be found. For the Metabolomics Workbench global study-level network, all mammalian studies with at least 100 annotated metabolites were selected for inclusion.

### Differential abundance testing

**Fig. 1:**
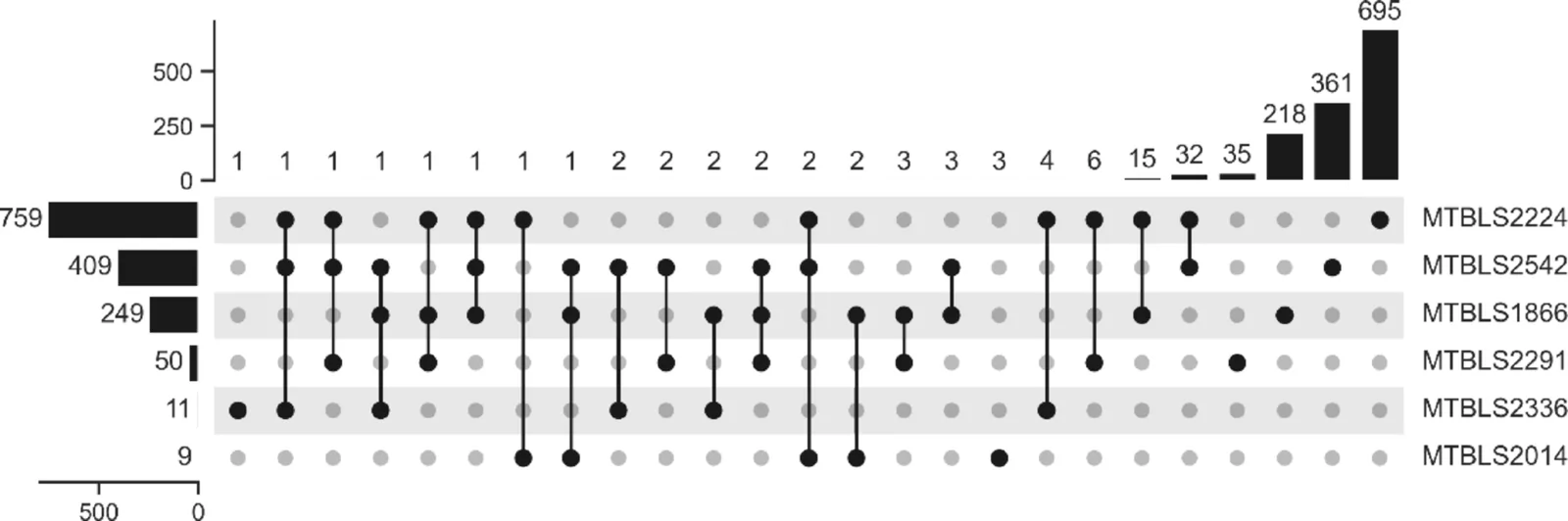
UpSet plot of differentially abundant (DA) metabolite co-occurrences in six COVID-19 studies from MetaboLights. The y-axis bar chart shows the number of differential metabolites in each of the six studies, whereas the x-axis bar chart shows the number of differential metabolites which co-occur across different combinations of the studies

**Fig. 2:**
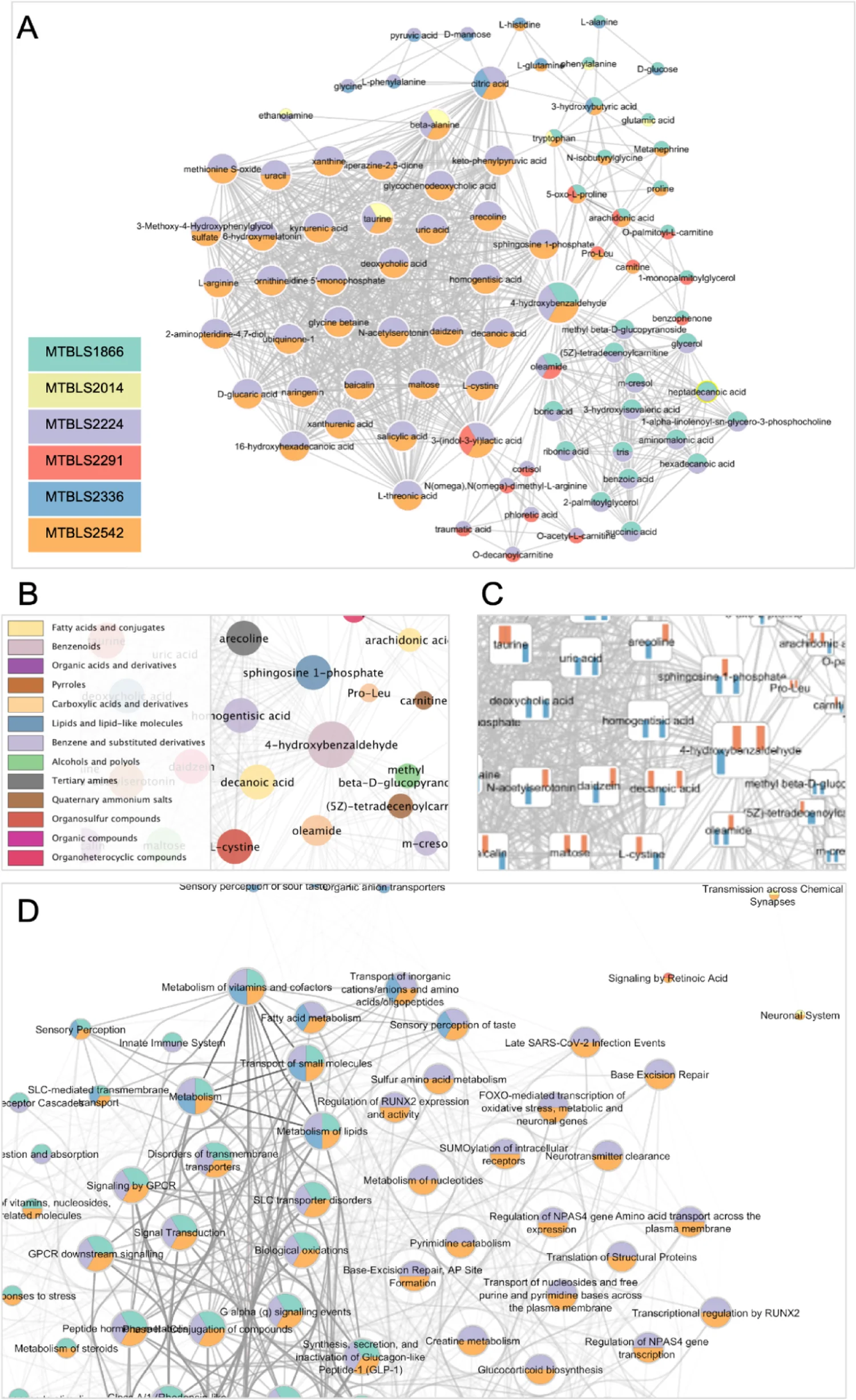
**A** COVID metabolite-level meta network. Nodes represent differential metabolites and edges represent co-occurrences of differential metabolites in studies. Only metabolites co-occurring in at least two studies are shown. Node size corresponds to degree. **B** Example nodes coloured by ClassyFire chemical class ontology. **C** Example nodes with bar chart graphic representing differential abundance (t-statistic) direction per study. **D** COVID pathway-level meta network. Nodes represent differential Reactome pathways and edges represent co-occurrences of pathways in studies. Only pathways co-occurring in at least two studies are shown. Node size corresponds to pathway coverage (number of metabolites mapping to a pathway). Organic edge router applied to enhance label visibility. For both panels A and D, pie chart colour represents study identity

**Fig. 3:**
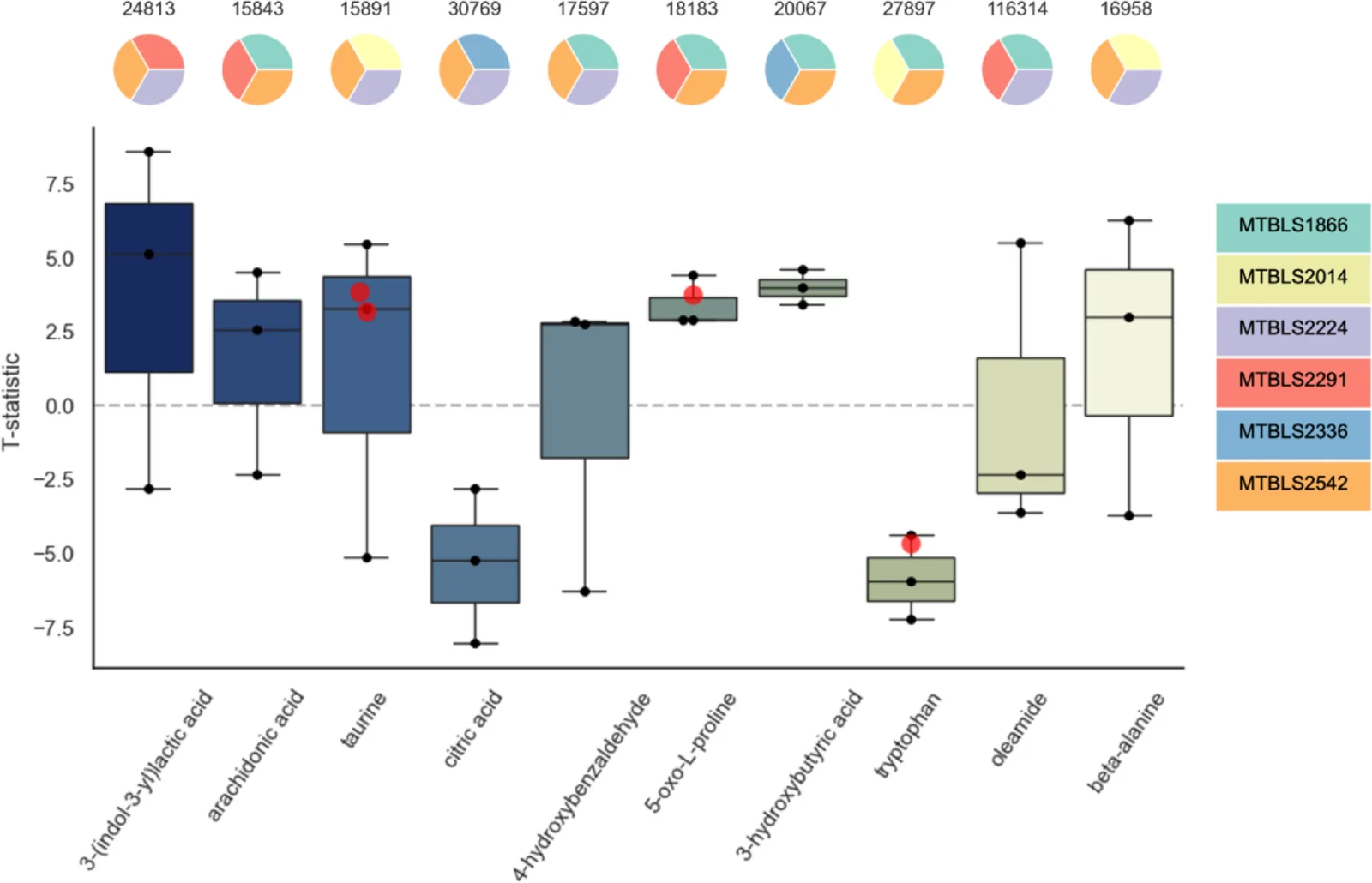
Validation of metabolite-level meta network using two additional studies (MTBLS6739 and MTBLS3852). Boxplots show t-statistics of metabolites that co-occur in at least 3 studies, representing the magnitude and direction of differential abundance. Red dots indicate metabolites present in validation studies. Pie charts indicate which studies contributed abundance measurements for each metabolite. ChEBI IDs are displayed above each pie chart

Differential abundance testing was performed at either the metabolite or pathway level using two-sided t-tests (scipy v1.13.1). At the metabolite level, each ChEBI identifier was assigned a p-value and Benjamini-Hochberg FDR-adjusted p-value based on a t-test between case and control groups. When performing differential abundance testing on LAMP outputs (see below), t-tests were performed on the abundance values for each annotated feature, and all ChEBI identifiers associated with features having significant p-values were considered differential metabolites (Figs. 1, 2 and 3).

At the pathway level, the metabolite data was first transformed to a pathway-space embedding using the ssPA KPCA method (v1.0.0) (Wieder et al., 2022) and Reactome Human Pathways (Gillespie et al., 2022) (release 90). This approach transforms a matrix of metabolite abundances into a matrix of pathway pseudo-activity scores, by using the first principal components derived from the sub-matrix of metabolite abundances corresponding to each pathway. Each pathway score was then assigned a p-value and adjusted p-value, again based on a t-test between case and control groups. Metabolites or pathways were deemed significantly differential if the FDR-adjusted p-value was ≤ 0.05. This constitutes the differential subset, $\:{d}_{s}$.

### Metabolite and pathway-level bipartite and meta network construction

**Fig. 4:**
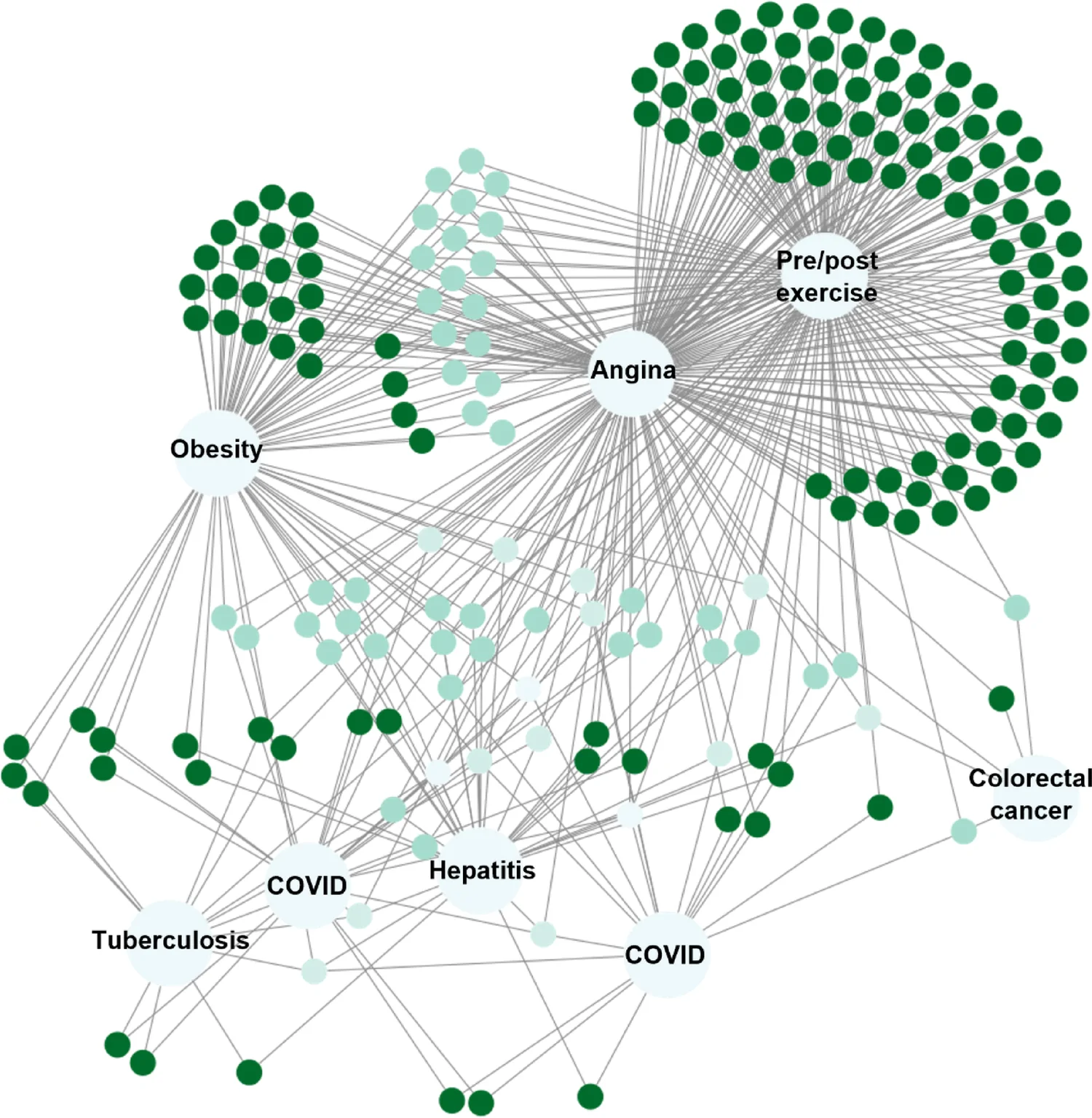
Metabolite-study bipartite network composed of studies focusing on different disease phenotypes. The bipartite graph linking differential metabolites to studies is visualised using a force-directed layout, such that diseases with similar metabolite profiles cluster together. Node colour reflects the degree of each node (darker nodes have lower degree i.e. number of connected edges)

*NetworkX* (v3.3) was used to create a bipartite graph $\:G=(U,\:S,\:E)$ consisting of vertices partitioned into two subsets: the studies $\:S=\left\{{s}_{1},\:{s}_{2},\:\dots\:,\:{s}_{N}\right\}$, and * U*, the set of all *D* metabolites differentially abundant in any study $\:U=\:\left\{{d}_{1},\:{d}_{2},\:\dots\:{d}_{D}\right\}$, connected by edges$\:\:E$. This graph can be represented by a bi-adjacency matrix * B* (size *N X D*) in which rows represent studies and columns represent differential metabolites, where $\:{B}_{ij}=1$ if metabolite *j* is differential in study *i*. This bipartite network was visualised in Fig. 4. To construct the meta network (e.g. Figure 3), a differential metabolite co-occurrence matrix $\:A={B}^{T}B$ (size *D X D*) was computed whose (*i*,* j*)’th element gives the number of studies in which both metabolites *i* and *j* are differential, with $\:{A}_{ij}\in\:[0,\:N]$. A value of 0 means a pair of differential metabolites do not co-occur in any study, whereas a value of 3 would mean this pair are differential in 3 studies. This matrix was transformed to an edge list (source node, target node, and weight) which was used as input to *NetworkX*. Node attributes, such as the study evidence contributions, direction of differential abundance, chemical class, and full chemical name were later added to the network representation for visualisation purposes. The same approach outlined above was applied to the network where *U* represents pathways instead of metabolites (e.g. for Fig. 3D).

### LAMP annotation

Prior to running LAMP (v1.0.3, https://github.com/wanchanglin/lamp*)*, XCMS (Smith et al., 2006) feature tables are required as the input. To this end, we re-processed the mzML files from the selected datasets from MetaboLights using the MetaboLights Labs Galaxy server (https://metabolights-labs.org/*).* To produce mzML, the raw vendor files must be converted. We note that different conversion tools may produce slightly different mzML outputs and this is a source of variability which users should be aware of. Only studies where mzML files were provided in MetaboLights could be used to run LAMP (see Table 2). Studies MTBLS2014, MTBLS2336, MTBLS2224, MTBLS2291, and MTBLS6739 could not be run either due to lack of uploaded mzML files or the use of NMR (in the case of MTBLS2336).

The XCMS pipeline involves the following functions, with parameters automatically detected based on the assay data such as chromatography column details: MSnbase readMSData, xcms findChromPeaks (xcmsSet) CentWave, xcms findChromPeaks Merger, xcms adjustRtime (retcor) Obiwarp, xcms groupChromPeaks (group).

The resulting XCMS feature tables and metadata were downloaded from the MetaboLights Galaxy Server API (Application Programming Interface), and used as the input to LAMP with default parameters (± 5 ppm mass window), and default reference (based on mammalian genome-scale metabolic models) and adduct library, specifying the appropriate ionisation mode. In instances where multiple ChEBI annotations were provided for a single XCMS feature, all ChEBI identifiers were used and added to the differential subset $\:{d}_{s}$. LAMP meta networks were created in the same way as the original networks, using the LAMP-generated ChEBI identifiers instead of the submitter annotations.

### Global study-level network construction

The Metabolomics Workbench API was used to download all RefMet annotations associated with each study (https://www.metabolomicsworkbench.org/tools/mw_rest.php*).* In addition, metadata from each study was downloaded, including assay details and sample metadata. A study by study adjacency matrix was created, where each value represents the number of overlapping RefMet identifiers between each pairwise combination of studies. This adjacency matrix was used to create a network using *NetworkX*, where nodes represent studies and edges represent the number of metabolites reported in pairs of studies.

### Network visualisation

Networks were visualised using *Cytoscape* (v3.10.2) and *Gephi* (v0.10.1). Within *Cytoscape*, the pie chart and bar chart node chart options were used to visualise study evidence contributions as well as direction of differential abundance. The ‘Edge Weighted Spring Embedded’ layout was used in *Cytoscape*, and the ‘ForceAtlas2’ was used in *Gephi*.

### Python notebooks and Dash app

To enable users to replicate the analysis shown here, Jupyter Notebooks and the demonstration datasets are provided on GitHub at: https://github.com/cwieder/MetaNetworkIntegration. In addition, to enable researchers to analyse their own and other public data, we provide an app implemented in Python (v3.12) using the Dash framework to provide an interactive web interface for integrative metabolomics data analysis. Data ingestion, preprocessing, and statistical analyses were performed using pandas, NumPy, SciPy, and related scientific computing libraries, with visualisation rendered via Plotly. Metabolite identifier harmonisation was supported through integration with the ChEBI 2.0 web API (Malik et al., 2026), enabling automated conversion between ChEBI identifiers and metabolite names. The app is available at https://github.com/EloisaRL/Metabolomic-data-analysis-app, where full documentation and usage instructions are provided. For reproducibility and cross-platform deployment, the application was containerised using Docker and distributed as a versioned Docker image.

## Results

### The metabolite-level meta network

The metabolite-level meta network represents differential metabolite co-occurrences across multiple studies. We demonstrated this approach using six COVID studies from the MetaboLights database (Table 1). All metabolomics data were profiled from plasma samples (five LC-MS and one NMR), and the study samples represented both healthy/asymptomatic and COVID patients of varying severities. Importantly the data integration was performed on common ChEBI IDs between studies, which ranged from 20 to 2,587 unique reported ChEBIs per study (Supplementary File 1). These ChEBIs were either provided by the submitter, or derived by MetaboLights using automated or manual pipelines during curation.

**Table 1:** MetaboLights studies used in the COVID-19 metabolite meta network

MetaboLights study ID (reference)	Purpose	Assay details	Number of ChEBI IDs (from the submitter)	Number of samples	Contrast	Biofluid
MTBLS2014 (Kimhofer et al., 2020)	Network	TQ RP+	33	49	Healthy/COVID	Plasma
MTBLS2291 (Aggarwal et al., 2021)	Network	LCMS RP+	934	143	Negative/Severe COVID	Plasma
MTBLS1866 (Barberis et al., 2020)	Network	GCxGC, LCMS RP+, LCMS RP-	476	96	Healthy/COVID	Plasma
MTBLS2224 (Zheng et al., 2021)	Network	GC, LCMS RP+, LCMS RP-	2,587	80	Healthy/COVID	Plasma
MTBLS2336 (Meoni et al., 2021)	Network	NMR	20	68	Healthy/COVID	Plasma
MTBLS2542 (Wu et al., 2021)	Network	LCMS RP+, LCMS RP-, Lipid RP+, Lipid RP-	503	70	Asymptomatic/Severe COVID	Plasma
MTBLS6739 (Le et al., 2023)	Validation	TQ RP+	39	200	Healthy/COVID	Plasma
MTBLS3852 (Ansone et al., 2021)	Validation	LCMS RP+	51	72	Healthy/Severe COVID	Serum

*TQ* triple quad, *RP* reversed phase chromatography, *LCMS* liquid chromatography mass spectrometry, *GCxGC* two-dimensional gas chromatography, *NMR* nuclear magnetic resonance spectroscopy, *HILIC* hydrophilic interaction liquid chromatography

Positive ionisation mode (+), Negative ionisation mode (-)

The bottom two studies are used for validation of the network

On each dataset, we performed t-tests to find metabolites which were differentially abundant (DA, FDR adjusted p-value ≤ 0.05) between non-COVID and COVID groups. Numbers of DA metabolites ranged from 9 to 759 across the six datasets. We recorded co-occurrences of each differentially abundant (DA) metabolite across the studies, visualised as an UpSet plot (Fig. 1). In general, the number of differential metabolite co-occurrences between studies was low, with just 10 DA metabolites co-occurring in at least three different studies, and no DA metabolites in common across 4, 5 or 6 studies (Fig. 1, Supplementary File 2). The low level of overlap in ChEBI IDs across the six studies was to be expected, as some studies had very few annotated ChEBIs to begin with, alongside likely discrepancies in the level of ChEBI ontology annotation assigned by the authors.

A bipartite graph was constructed, where each study is linked to its differential metabolites. The resulting metabolite-level meta network consisted of 82 nodes (differential metabolites) and 907 edges (differential metabolite co-occurrences in studies), after filtering for metabolites which co-occurred in at least two studies (Fig. 2A). Cytoscape was used to visualise the network, using the pie chart node graphic to represent which studies contributed differential metabolites (nodes). From Fig. 2A, it is clear that oleamide (CHEBI:116314) and 4-hydroxybenzaldehyde (CHEBI:17597), for example, were found as differentially abundant in 3/6 studies, and therefore may be metabolites of interest. Nodes can also represent other properties, for example chemical class, or direction of differential abundance of each metabolite per study (Fig. 2B and C). A pathway space representation of the network was produced, using single-sample pathway analysis (Wieder et al., 2022). Each COVID dataset was transformed to a pathway-level embedding (see Methods) and differential abundance testing was performed on Reactome pathway scores rather than metabolites. The pathway-level meta network is constructed in the same manner as its metabolite-level counterpart, where nodes represent differential pathways (Fig. 2D).

The integrated network can also be used to ‘validate’ findings from new studies, and determine whether there is correspondence in the differential metabolites and the direction of change. We obtained two additional COVID-19 studies (MTBLS6739 and MTBLS3852) which were not used to construct the initial network. We then investigated whether these studies agreed with the co-occurring differential metabolites found in the initial meta network. We focused on the 10 metabolites which co-occurred in at least 3/6 studies (Fig. 3). The boxplots in Fig. 3 show the t-statistic of each metabolite in each study, representing the metabolite’s overall direction and magnitude of differential abundance. The pie charts show which studies contributed to the differential abundance (same legend as Fig. 2A), demonstrating a diverse range of study contributions. Given the large variation in study sizes, effect sizes, sampling, patient populations and study designs, we expect some level of disagreement as illustrated by the box plots. Nonetheless, in the two validation studies, 1/10 and 3/10 metabolites were also differentially abundant, and encouragingly, the direction of the t-statistic appears to correlate well with the prior studies, demonstrating a good level of agreement. 5-oxo-L-proline (CHEBI:18183), for example, could be considered a robust differential feature in COVID-19, as it was found to be differential in 3/6 studies and an additional validation study. Furthermore, the direction of differential abundance (positive t-statistic) in four studies indicates this putative biomarker will likely be found elevated in COVID-19 plasma samples.

The level of disagreement across studies is harder to quantify, as our approach is based on shared ChEBI IDs only. Metabolites which show few differential co-occurrences across studies may either not be robust putative biomarkers, or simply may not have been profiled (or not annotated at the same level of ChEBI specificity).

The network can be extended to more than just studies of a related phenotype. It may be of interest to visualise how studies with different contrasts compare to each other in terms of their differential metabolite signatures. As an example, we integrated Metabolomics Workbench studies on COVID-19, hepatitis, tuberculosis, obesity, angina, colorectal cancer, and exercise effects (Table S1). In this case, as we used Metabolomics Workbench studies, the mapping between studies takes place using RefMet identifiers, rather than ChEBI IDs. Figure 4 shows the bipartite graph layout linking differential metabolites (irrespective of direction of association) in each study to the study nodes. The force-directed layout reveals clustering patterns which are consistent with biological interpretation, for example COVID studies cluster together, as do those which are inflammatory in nature (hepatitis, tuberculosis), and those which relate to the cardiovascular system (angina, pre/post exercise changes).

### The impact of harmonised annotation on data integration

As we are integrating metabolomics datasets based on a harmonised identifier (i.e. ChEBI or RefMet), the resulting networks are highly dependent on the number of compound assignments available in a dataset provided by either the submitter or automated annotation pipelines. Many submitted studies only include compound assignments for a small proportion of detected metabolites, typically those reported as statistically significant for the biological question of interest to the submitters.

To increase the number of available ChEBI compound assignments in the COVID-19 case study with all data derived from MetaboLights, we applied the Liverpool Annotation of metabolites using Mass sPectrometry (LAMP) pipeline (Lin, 2025). LAMP uses a manually curated reference library derived from mammalian genome-scale metabolic models to annotate LC-MS data via exact mass, multiple adduct search, and adduct correlation profiles from feature tables. The resulting annotations, while tentative, are more consistent than those derived from original submitters who used differing and often undocumented annotation methods. Nonetheless, some false positive or negative annotations might be expected as the price paid for this harmonised annotation approach. Using LAMP we re-annotated all applicable COVID-19 studies (Table 2), which increased the number of ChEBI identifiers per study. For example, MTBLS1866 contained 211 ChEBIs from the submitter, but after LAMP obtained 837 ChEBI annotations. 5 of the 8 studies were not amenable to LAMP annotation due to lack of appropriate peak list and intensity data files in mzML format. In the three applicable studies, on average per study, the numbers of total and differential ChEBIs increased by factors of ~ 6 and ~ 7 respectively. The median number of ChEBIs per annotated XCMS feature ranged from 1 to 2 per study, indicating only a moderate degree of annotation ambiguity. We used these new sets of ChEBI annotations (or originally submitted ChEBI IDs where LAMP could not be run due to absence of mzML files provided) to re-compute the metabolite-level meta network. We compared the resulting network to the metabolite-meta network generated with the original data.

**Fig. 5:**
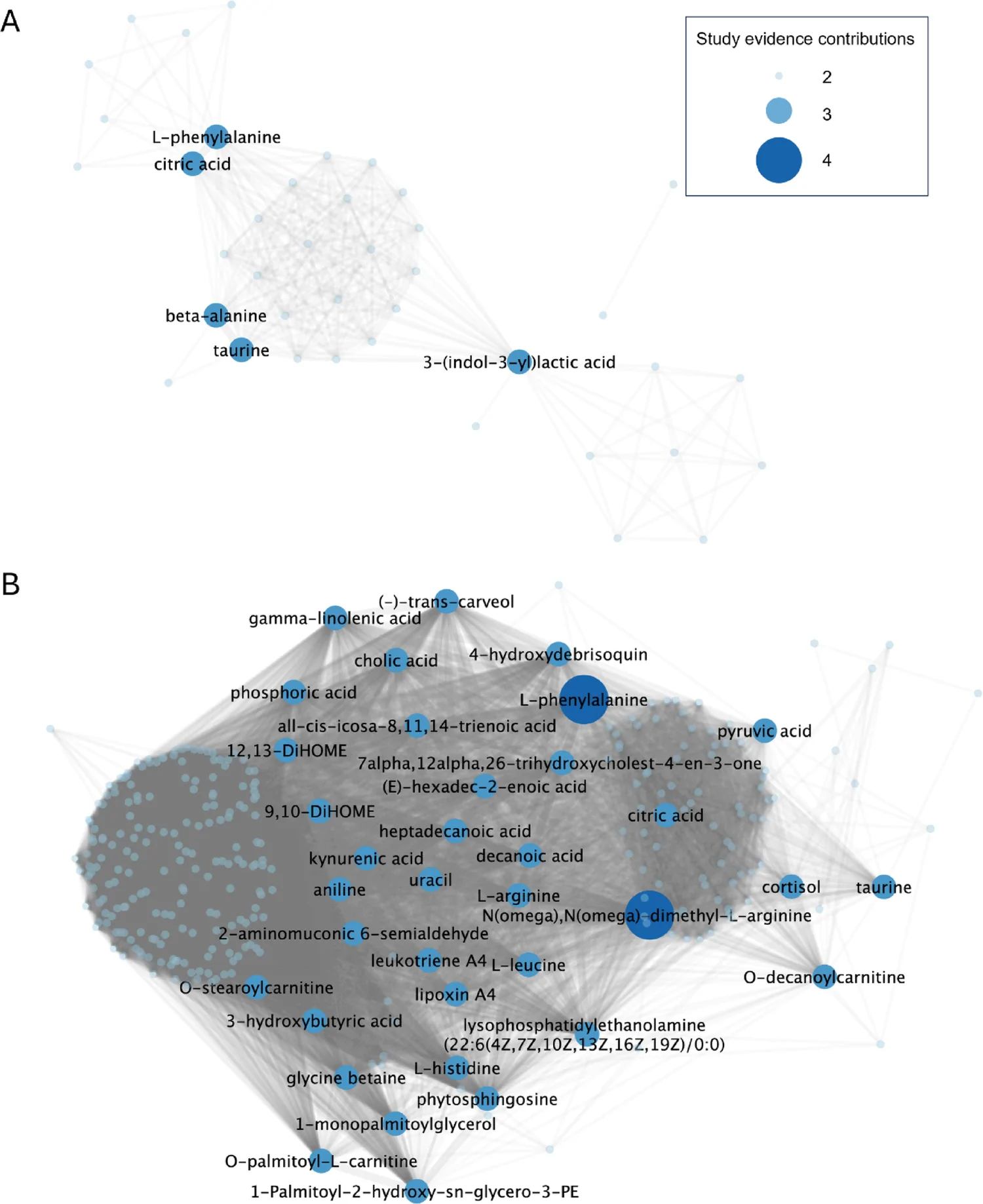
Metabolite-level meta network before and after applying LAMP annotation to the six original studies. Nodes represent differential metabolites and edges represent differential metabolite co-occurrences in studies. **A** Metabolite-level meta network using submitter ChEBI annotations (excluding assays not used in (**B**)). **B** Metabolite-level meta network using combined LAMP and submitter annotations. Node size and colour represent the number of study evidence contributions. Edges represent co-occurrences between differential ChEBI IDs. Nodes with at least 3 study evidence contributions are labelled

There were 40 nodes (differential metabolites) in the original meta network, compared to 320 nodes in the LAMP meta network, with 32 differential ChEBIs in common (90% of nodes were new). Figure 5A shows the network created using submitter-provided annotations, and 5B using LAMP augmented annotations. As expected, using more annotations resulted in a much denser network. Using LAMP annotations, the network contained nodes that had up to four study evidence contributions, compared to a maximum of three in the original network. L-phenylalanine for example had three study evidence contributions in the original meta network, which increased to four with the use of LAMP. The increased study evidence contributions can strengthen the confidence of a metabolite as a feature of interest, and the use of LAMP annotations shows this is highly dependent on having a harmonised set of identifiers as input. We were able to annotate one of the ‘validation’ studies using LAMP (MTBLS3852), and used this alongside MTBLS6739 to compare the differential signature of metabolites that co-occurred in at least three studies used in the LAMP meta network (Figure S1). In general the validation studies showed weak to little evidence of concordance with the original studies after LAMP annotation, perhaps partly due to increased uncertainty in compound annotations. Indeed, it should be highlighted that LAMP only uses mass (*m/z*) of the intact metabolite with a specialised library for compound assignment and use of retention time and fragmentation (not possible in this case) is recommended to increase compound assignment confidence.

**Table 2:** LAMP annotation of COVID-19 studies

MetaboLights study ID	Number of ChEBI IDs (from submitter)	Number of ChEBI IDs (after LAMP)	Number of differential ChEBI IDs	Number of differential ChEBI IDs (after LAMP)	Median number of ChEBI annotations per XCMS feature (after LAMP)
MTBLS2014	33	NA	9	NA	NA
MTBLS1866 (+ mode only)	211	837	128	262	Median = 1, IQR = 1
MTBLS2336	20	NA	11	NA	NA
MTBLS2542 (- mode only)	296	1,639	177	1,272	Median = 2, IQR = 2
MTBLS2224	2,586	NA	759	NA	NA
MTBLS2291	130	NA	50	NA	NA
MTBLS6739	39	NA	27	NA	NA
MTBLS3852	51	456	20	243	Median = 1, IQR = 1

Studies that did not contain suitable input peak list and intensity data files in mzML format for running LAMP are indicated by NA (after LAMP)

### The global study-level network

While the metabolite-level meta network (and its pathway-space counterpart) are useful visualisations for the interpretation of metabolite effects in groups of studies, a more global approach can offer an intuitive view of an entire database, for example insights into study similarity and assay/metadata coverage. Here we present a study-level network which gives a global overview of studies in the Metabolomics Workbench repository. Instead of focusing on differentially abundant metabolites as we did in the metabolite-level meta network, studies were linked by all metabolites profiled and reported. The preferred reference system for Metabolomics Workbench studies is the RefMet nomenclature, consisting of over 600,000 chemical names, many of which are linked to class information from ClassyFire (Djoumbou et al., 2016) or LipidMaps (Conroy et al., 2024).

**Fig. 6:**
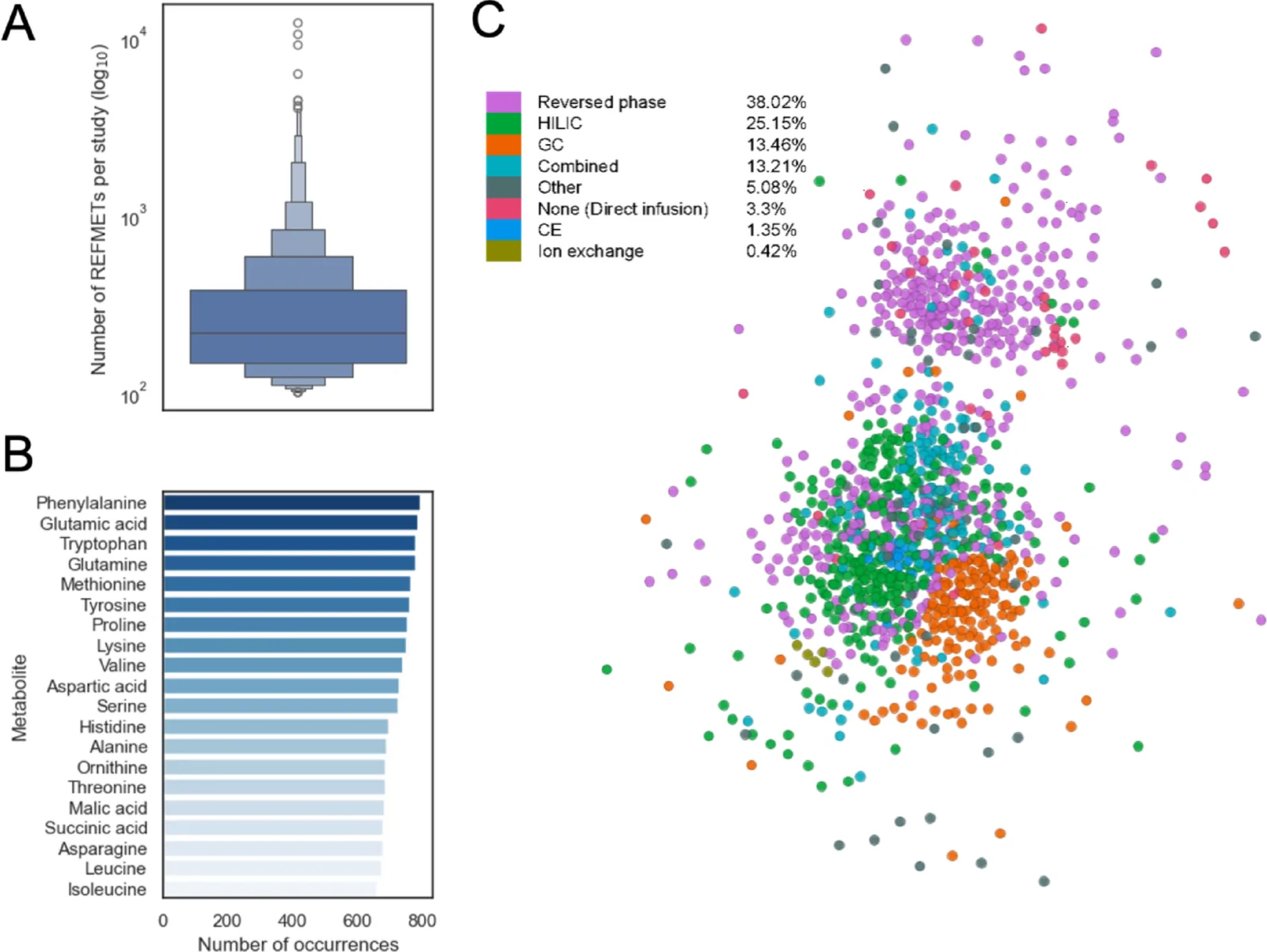
**A** Number of RefMet metabolite annotations per study in Metabolomics Workbench as of Dec 2024. **B** Top 20 most frequently annotated metabolites across all studies in Metabolomics Workbench. **C** study-level metabolite co-occurrence network for the Metabolomics Workbench repository visualised using a force-directed layout. Nodes represent studies linked by edges (not shown for clarity), which represent the number of shared metabolites

Using the Metabolomics Workbench API, we extracted all named metabolites (in RefMet format) for each of 1,181 studies which reported at least 100 named metabolites. All studies were of mammalian origin but corresponded to different organisms and sample types. An adjacency matrix was created where each pair of studies was linked by the number of common RefMet metabolites profiled. The median number of annotated RefMet metabolites per study was 221 (mean = 346) (Fig. 6A), while the top 20 metabolites most often profiled across all studies were amino acids and organic acids involved in central carbon metabolism (Fig. 6B). Study properties can be visualised on the network representation, for example disease, or assay technique. Figure 6C shows the resulting global study-level network for Metabolomics Workbench, with study nodes coloured by assay technology. Encouragingly, nodes broadly cluster according to assay type, suggesting the metabolites profiled using each technology tend to be distinctive. Reversed-phase studies were represented in two clusters which probably represent lipidomic-based studies (top cluster) and aqueous reversed-phase studies focused on water-soluble metabolites (bottom cluster) because water-soluble metabolites will also be detected by HILIC and GC assays which co-cluster.

## Discussion

Here, we present a novel approach for integrating metabolomics data from large-scale public data repositories, along with a Python based web-app implementing the method. Using networks, we promote the simultaneous re-use and integration of multiple study datasets either to address a specific research question (metabolite/pathway-level meta network) or obtain an exploratory database-wide view (global study-level network). The metabolite-level meta network allows users to select a specific subset of studies from a repository and integrate them based on common differential metabolites. This approach enables easy visual identification of differential metabolites (represented as ChEBI identifiers) that co-occur across multiple studies. In this paper and the associated Python web app, we focus on metabolites differential between two sample groups, but the methodology is applicable to almost any study design. For example, metabolites could be selected as differential based on associations with continuous or survival outcomes. The pathway-level meta network enables similar analysis, instead using higher-level biological processes which can further aid the interpretation. Users can then pinpoint putative biomarkers or pathways of interest to further investigate using laboratory experiments.

Using COVID-19 metabolomics studies deposited in the MetaboLights repository as a case study, we produced both metabolite and pathway-level meta networks. For six studies with similar disease state contrasts (e.g. control vs. COVID, or mild vs. severe COVID), the approach allowed us to integrate data from a diverse array of assay technologies, including both targeted and untargeted MS and NMR workflows. We were able to ascertain 10 metabolites which showed differential metabolite co-occurrence in three out of six studies. These were 3-(indol-3-yl)lactic acid (CHEBI:24813), arachidonic acid (CHEBI:15843), taurine (CHEBI:15891), citric acid (CHEBI:30769), 4-hydroxybenzaldehyde (CHEBI:17597), 5-oxo-L-proline (CHEBI:18183), 3-hydroxybutyric acid (CHEBI:20067), tryptophan (CHEBI:27897), oleamide (CHEBI:116314), and beta-alanine (CHEBI:16958). Arachidonic acid, taurine, and tryptophan have well-characterised roles in immune-metabolism and inflammatory pathways (Badawy, 2023; Khoramjoo et al., 2024; Ripon et al., 2021). The kynurenine pathway of tryptophan metabolism has been associated with COVID-19 pathology, with depletion of serum tryptophan levels often associated with worsening severity or long-COVID outcomes (Al-Hakeim et al., 2023; Badawy, 2023; Essex et al., 2024). Encouragingly, the COVID-19 metabolite-level meta network revealed a depletion of tryptophan across four different studies, highlighting its relevance as a potential therapeutic target. Other potential markers are less studied within the COVID-19 literature, but could provide promising avenues for further research. Oleamide for example is an endocannabinoid lipid molecule (Mendelson & Basile, 2001) which has recently been associated as a marker of COVID severity and plays a role in neuromodulation (Byeon et al., 2022; Delafiori et al., 2024). Finally, using two additional COVID-19 studies from MetaboLights, we performed differential abundance testing and compared both magnitude and effect size of the t-statistic of metabolites present in the original meta network. Encouragingly, of those metabolites that had matching ChEBI IDs (four metabolites across two studies), all had similar magnitude and direction of change as those in the original network.

To date there are few available tools that focus on integrative visualisation of entire metabolomics data repositories or a selected subset of studies (Choudhury et al., 2020; Pang et al., 2024; Schmid et al., 2023; Wang et al., 2016; Yurekten et al., 2024). Our global study-level network offers a solution to this, where each node represents a metabolomics study in a repository, connected by similarity of assayed metabolites. Colouring nodes by various metadata, such as chromatography technology, instrument, etc., can identify trends at the repository scale. It is important to consider the quality of the input study data used to construct such networks. For example, each study will be annotated differently, resulting in either very few or perhaps very many (1000’s of) annotations. The level and quality of metadata provided will also impact the results and the level of insight users may glean from them.

The integration of metabolomics data was made possible via the use of a harmonised identifier: in this work ChEBI and RefMet were used as a reference system. However, our work demonstrates that repository studies often have limited annotations (either provided by the submitter or that could be mapped to a reference system), which constrains the integration possible. To this end, we employed the LAMP pipeline to re-analyse the COVID studies where possible, providing a much larger number of ChEBI IDs on which to perform the integration, and harmonising the workflow by using the same software and reference system. However, it is important to note that LAMP annotates features based on MS1 data only, i.e. using exact masses within a specified ppm window, yielding a level 3 annotation (Sumner et al., 2007). This does not require orthogonal information such as MS/MS, and so can be applied to a wide range of studies in public repositories. This approach can yield false positive annotations as it cannot distinguish e.g. between isobaric species (Kind & Fiehn, 2006). Nonetheless, the manually curated LAMP reference file and adduct library employ expert domain knowledge to reduce such false positive matches. This example illustrates the trade-off between more confident but heterogeneous annotations from submitters (who may have access to information beyond that stored in the repository), and less confident but highly consistent annotations via harmonised approaches (e.g. LAMP). Where a harmonised approach is used, the effect on the meta networks is that there are many more nodes and edges, meaning that more co-occurrences between inter-study differential metabolites can be found. Increasing numbers of study evidence contributions to nodes can also help improve confidence in the annotations. Abstracting the network to the pathway level can potentially increase confidence further, as metabolites are combined into manually curated functional groups, emphasising biological signal and potentially minimising noise caused by false positive annotations (Wieder et al., 2024). Future work could incorporate filtering based on annotation confidence, where reported (e.g. using Metabolomics Standards Initiative levels). One could also make use of the ChEBI ontology to construct the networks using parent terms, thereby unifying more granular ChEBI IDs and harmonising across studies further.

## Conclusion

Here, we have presented an integrative, network-based approach for the joint analysis of multiple metabolomics datasets from public repositories. The method is implemented in a Python based web app which is freely available. Direct from the repository, datasets can be integrated, analysed, and visualised using a network representation where users can intuitively examine patterns and trends amongst studies and metadata. This work was limited by the quality of the submissions in public repositories, especially the quality and quantity of annotations reported by the submitters. Harmonised annotation using LAMP increased the consistency and number of annotations, deepening the richness of the resulting networks. In the future, we envision that data repositories will benefit from automated re-annotation pipelines that also make use of orthogonal data such as retention time, as well as any MS2 data from submitters, to obtain as accurate compound annotations as possible. The pipelines presented here can also be implemented directly within database web services, enabling users to easily select relevant studies, construct integrated networks, and compare with their own data.

## Electronic Supplementary Material

Below is the link to the electronic supplementary material.

Supplementary Material 1

Supplementary Material 2

Supplementary Material 3

## Data Availability

All data used in this study is publicly available on the MetaboLights and Metabolomics Workbench repositories as detailed in the article, Table 1 & Table S1.

## Code availability

Jupyter Notebooks containing the code used to generate the results of this work as well as a demonstration dataset are available on GitHub at: https://github.com/cwieder/MetaNetworkIntegration. The interactive Dash app alongside detailed installation guide and tutorials are available freely at https://github.com/EloisaRL/Metabolomic-data-analysis-app/tree/main.

## Declarations

### Research involving human and animal participants

This article does not contain any studies with human and/or animal participants performed by any of the authors.
