# Supplementary material for: Network-based integration of metabolomics data from large-scale repositories
Source: Metabolomics. 2026 Jul 15;22(4):127. doi: 10.1007/s11306-026-02507-4 (PMC13372841; doi:10.1007/s11306-026-02507-4)
Supplement: Supplementary file 3 — Supplementary Material 3 [file 11306_2026_2507_MOESM3_ESM.docx]

Supplementary Information

Network-Based Integration of Metabolomics Data from Large-Scale Repositories

Cecilia Wieder^1^, Eloisa Rocha Liedl^1^, Thomas Payne^2^, Ozgur Yurekten^2^, Callum Martin^2^, Felix Xavier Amaladoss^2^, Noemi Tejera Hernandez^2^, Wanchang Lin^3^, Yasin El Abiead^4^, Pieter C Dorrestein^5^, Claire O’Donovan^2^, Juan Antonio Vizcaíno^2^, Warwick Dunn^3^, Timothy Ebbels^1^*

1. Section of Bioinformatics, Division of Systems Medicine, Department of Metabolism, Digestion & Reproduction, Imperial College London, Hammersmith Hospital, Du Cane Road, London W12 0NN, UK
2. European Molecular Biology Laboratory, European Bioinformatics Institute (EMBL-EBI), Wellcome Trust Genome Campus, Hinxton, Cambridge CB10 1SD, UK
3. Centre for Metabolomics Research, Department of Biochemistry, Cell and Systems Biology, Institute of Systems, Molecular and Integrative Biology, University of Liverpool, Liverpool, L69 7ZB, UK
4. BOKU University, Institute of Analytical Chemistry, Department of Natural Sciences and Sustainable Resources
5. Skaggs School of Pharmacy and Pharmaceutical Sciences, University of California, San Diego, La Jolla, CA 92093

*Corresponding author: [t.ebbels@imperial.ac.uk](mailto:t.ebbels@imperial.ac.uk)

**Table S1: Studies from Metabolomics Workbench used to construct the cross-phenotype metabolite-level meta network of Figure 4.**

| Metabolomics Workbench study ID (publication)* | Disease/outcome | Assay | Sample size | Number of REFMET annotations |
| --- | --- | --- | --- | --- |
| ST002016 (N/A) | COVID | MS, RP+, RP- | 95 | 424 |
| ST002301 (N/A) | COVID | MS, RP+ | 149 | 93 |
| ST000284 (Zhu *et al.*, 2014) | Colorectal cancer | TQ MS | 147 | 103 |
| ST002428 (N/A) | Tuberculosis | MS RP+, MS RP- | 43 | 70 |
| ST002100 (N/A) | Hepatitis | MS RP+, MS RP- | 36 | 68 |
| ST001412 (N/A) | Obesity | MS RP+ | 86 | 668 |
| ST001789 (N/A) | Response to exercise | MS HILIC, MS RP | 37 | 439 |
| ST001420 (N/A) | Angina | TQ MS | 747 | 391 |

* Publication linked to dataset in Metabolomics Workbench, N/A if not available.

**Figure S1:** Comparison of metabolite-level meta network results with two additional studies (MTBLS6739 using original annotations and MTBLS3852 enhanced with LAMP annotation). Boxplots show t-statistics of metabolites that co-occur in at least 3 studies, representing the magnitude and direction of differential abundance. Red dots indicate metabolites present in validation studies.
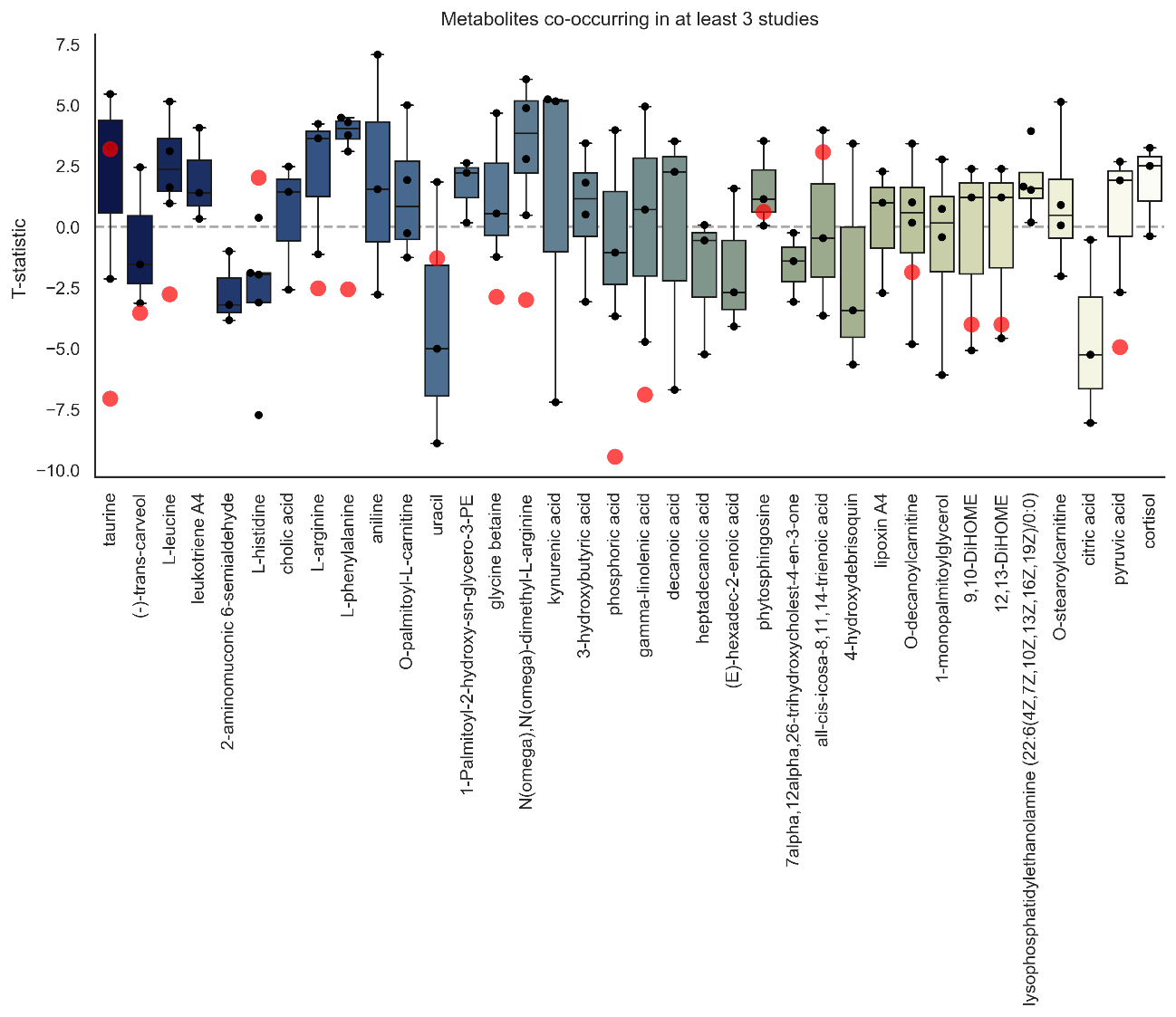


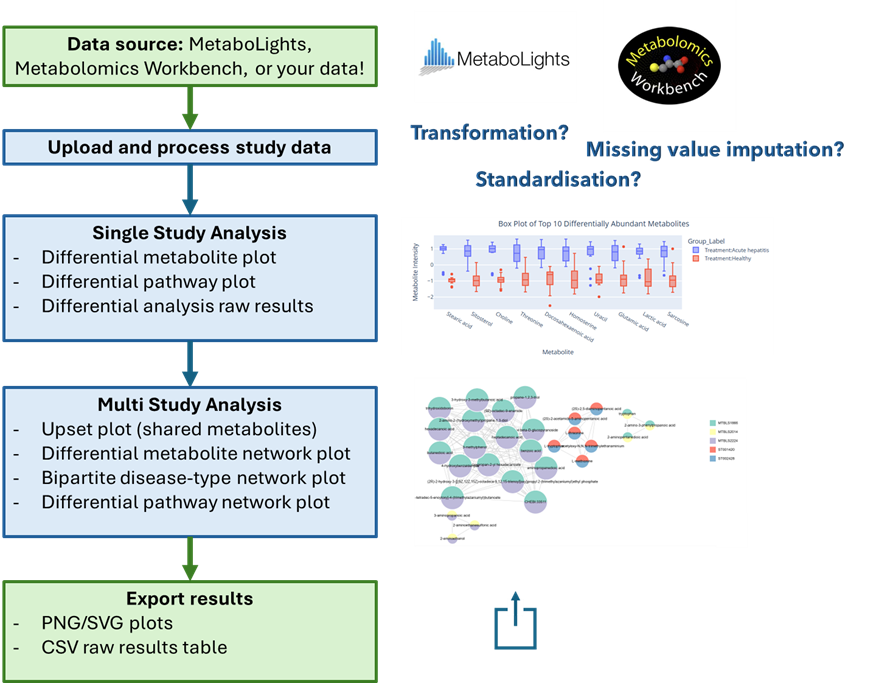


**Figure S2: Workflow of the metabolomics data analysis app.** Metabolomic datasets are uploaded from public repositories (MetaboLights, Metabolomics Workbench) or provided by the user. The data are then processed using configurable methods for missing value imputation, transformation, and standardisation. The application supports both single-study analyses and comparative multi-study analyses, generating differential metabolite and pathway results, as well as network-based visualisations. All outputs can be exported as publication-ready plots (PNG/SVG) or as raw results tables (CSV). The app is available at <https://github.com/EloisaRL/Metabolomic-data-analysis-app/tree/main>.

**References**

Zhu, J., Djukovic, D., Deng, L., Gu, H., Himmati, F., Chiorean, E.G. and Raftery, D. (2014) Colorectal Cancer Detection Using Targeted Serum Metabolic Profiling. *Journal of Proteome Research* **13,** 4120-4130.
